# Supplementary material for: Modeling Tick Populations: An Ecological Test Case for Gradient Boosted Trees
Source: bioRxiv. 2023 Mar 24:2023.03.13.532443. Preprint. [Version 2] doi: 10.1101/2023.03.13.532443 (PMC10054924; doi:10.1101/2023.03.13.532443)
Supplement: Supplementary file 1 [file NIHPP2023.03.13.532443V2-supplement-1.pdf]

bioRxiv preprint doi: https://doi.org/10.1101/2023.03.13.532443; this version posted March 24, 2023. The copyright holder for this preprint (which was not certified by peer review) is the author/funder, who has granted bioRxiv a license to display the preprint in perpetuity. It is made available under aCC-BY 4.0 International license.

Appendix A: Supplementary data

Supplemental Table 1: Most Predictive Ecological Features from Gradient Boosted Occurrence and Abundance Models compared to Linear Counterparts

**Model**	GBM Occurrence	GLM Occurrence	GBM Abundance	GLM Abundance
**Physical Habitat**	Longitude (+, NL), Distance to nearest road (-, NL)	Latitude (+), Elevation (-), Distance to nearest road (+), Road type of nearest road (NL), Indicator of critical zone (-)	Latitude (-, NL), Longitude (+, NL)	Latitude (-), Longitude (+), Elevation (NL), Forest (-), Distance to nearest hydrography feature (-)
**Vapor Pressure**	Maximum Jan 2 years prior (-, NL), Minimum Oct 2 years prior (NL), Maximum Oct 1 year prior (+, NL), Maximum Jan (-, NL), Minimum June (-, NL), Minimum October (+, NL)	Minimum Jan 1 year prior (-)		Maximum October 2 years prior (+), Minimum October 2 years prior (-, NL)
**Temperature**	Mean differential Jan 2 years prior (+, NL, IE), Degree days above 0 C spring-summer 1 year prior (+, NL, IE), Degree days above 0 C spring 1 year prior (+, NL), Maximum June 1 year prior (+, NL, IE)	Degree days above 0 C spring 2 years prior (-), Degree Days below 0 C winter 1 year prior (+), Degree days above 0 C spring-summer 1 year prior (+)		Degree days above 0 C spring-summer 1 year prior (+)
**Day of Collection**	Person-hours collecting (+, NL), Month (NL)	Person-hours collecting (+), Month (NL), Local Temperature (+), Wet (-)	Person-hours collecting (+, NL), Week (NL)	Person-hours collecting (+), Month (NL)
**Miscellaneous**		Deer harvest (-)	Deer harvest (NL)	Deer harvest (+)

Top 15 most predictive features from the gradient boosted occurrence model and all features from the other models are included.
(-) = negative relationship, (+) = positive relationship, NL = nonlinear relationship, IE = interaction effect

Supplemental Data 1: Table containing all features used by the gradient boosted models can be found at: MendeleyData (doi: https://doi.org/10.17632/w8bp678m3f.2).
